# Discharge Behavior and Dielectric Breakdown of Oxide Films during Single Pulse Anodizing of Aluminum Micro-Electrodes

**DOI:** 10.3390/ma12142286

**Published:** 2019-07-17

**Authors:** Kai Yang, Haisong Huang, Jiadui Chen, Biao Cao

**Affiliations:** 1 Key Laboratory of Advanced Manufacturing Technology of the Ministry of Education, Guizhou University, Guiyang 550025, China; 2 College of Mechanical and Automotive Engineering, South China University of Technology, Guangzhou 510640, China

**Keywords:** plasma electrolytic oxidation, electrical characteristic, anodizing, SEM, aluminum

## Abstract

Micro-arc discharge events and dielectric breakdown of oxide films play an important role in the formation process of plasma electrolytic oxidation coating. Single pulse anodization of micro-electrodes was employed to study the discharge behavior and dielectric breakdown of oxide films deposited on aluminum in an alkaline silicate electrolyte. Voltage and current waveforms of applied pulses were measured and surface morphology of micro-electrodes was characterized from images obtained using scanning electron microscope (SEM). A feasible identification method for the critical breakdown voltage of oxide film was introduced. Different current transients of voltage pulses were obtained, depending on applied pulse voltage and duration. In addition, the active capacitive effect and complex non-linear nature of plasma electrolytic oxidation process is confirmed using dynamic electrical characteristic curves. A good correlation between the pulse parameters and shape of discharge channels was observed. Circular opened pores were found to close with increasing potential and pulse width. Finally, the characteristic parameters of a single discharge event were estimated.

## 1. Introduction

Plasma electrolytic oxidation (PEO), often also known as micro-arc oxidation (MAO), is a novel environmentally friendly surface modification technique used for the production of ceramic coatings on a variety of light metals, such as Al, Mg, Ti and their alloys [1,2]. PEO is a complex and highly non-linear process due to the electrical, thermal, and plasma-chemical reactions in the electrolyte. Over the last decade, some prominent researches have been reported in literature related to the formation mechanism of PEO coatings. Various approaches have been reported in literature to identify the mechanism for the formation of the coating due to PEO, such as thickness measurement [3], element tracer [4,5], two-step oxidation method [6], and optical emission spectroscopy (OES) [7]. Nevertheless, it is still difficult to explain such a complex physical and chemical process during plasma discharge and significant work still needs to be undertaken to explain the formation mechanism of PEO coatings.

During PEO, dielectric breakdown of the growing oxide coatings at high voltage electrolysis results in a large number of short-lived micro-discharges [8]. Those discharge events mean not a single discharge but a cascade of smaller discharges that are bundled together in space and time. These distinct discharge events play an important role in the formation mechanism of the coating and strongly affect the microstructure and properties of the coating [9]. Due to the extreme non-linearity associated with the plasma discharge, monitoring the evolution of an individual discharge event would aid in understanding the coating mechanism and allow optimization of the production process.

Recently, many methods, such as optical [10,11,12], spectral [12,13,14,15,16], electrical [16,17], frequency response [18,19], and acoustic [20], have been used to measure dynamic parameters of plasma discharge during PEO. Reported characteristic parameters, including duration, current level, apparent radii, event rate, temperature, and electron density, about PEO discharge events are summarized [10,11,12,13,14,15,16,17,21] as follows: Typical discharges are now known to occur in prolonged sequences (‘cascades’) at particular locations, and to have lifetimes of the order of a few tens to a few hundreds of microseconds, with ‘incubation’ periods between them of around a few hundred μs to a ms or two. Discharge currents are typically several tens of mA, discharge energies a few mJ and diameters of core discharge channels a few tens of μm. The frequency of plasma discharges per unit time and area was determined throughout processing, falling from initial values in the range 300–350 mm^−2^ s^−1^ to fewer than 50 mm^−2^ s^−1^ after 1000 s of processing. Plasma temperatures have been estimated via optical spectroscopy to range from about 4000 to 12,000 K, with some indications of a higher temperature core and a lower temperature surrounding region. Corresponding electron densities typically range from ~10^15^ to 10^18^ cm^−3^. 

It can be seen that these monitoring techniques are effective in distinguishing certain basic characteristics of PEO discharge events. However, values of these characteristic parameters are spread across a wide range, sometimes differing by several orders of magnitude. For example, the lifetimes of discharges revealed by fast video imaging were in the range of 0.05–4 ms during AC PEO of magnesium alloys [10] and evaluations based on the spectroscopic method for the Al 1100 alloy show electron temperatures to be in the range of 3500–9000 K for a unipolar current mode and in the range of 3500–6000 K for a bipolar current mode [14]. This is probably the result of a low-resolution tool or inappropriate measurement procedure and data processing algorithm.

Special experimental methods, such as small area in-situ testing [16] and single pulse anodizing [22,23,24], have been designed to study discharge events and dielectric breakdown during PEO. The present work builds upon single pulse anodizing of aluminum micro-electrodes. Here, a partial aim is to produce a clear set of conclusions related to the discharge behaviors. In addition, an improved understanding of the effect of plasma discharge on subsequent oxide film formation is sought. Electrical characteristic monitoring and scanning electron microscopy (SEM) were used to study the effect of single pulse anodizing on aluminum micro-electrodes along with the effects of pulse parameters on surface morphology. Parameters such as current level, duration, diameter, and spatial distribution ratio were estimated from the current waveforms.

## 2. Materials and Methods 

### 2.1. Materials and Pre-Treatment

**Figure 1:**
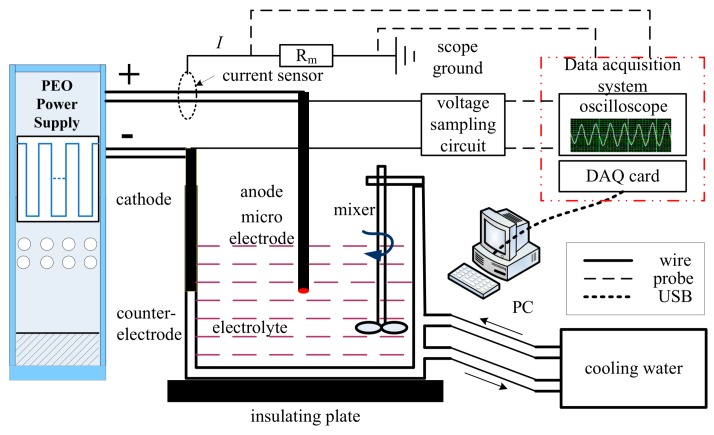
Schematic of the experimental platform for plasma electrolytic oxidation (PEO).

The experimental platform used to study PEO is shown in Figure 1. A home-made 20 kW pulse power supply was used, which could provide a DC voltage ranging from 0 to 1000 V and pulses with the same magnitude and frequencies up to 20 kHz [25]. In this study, small area sample monitoring of currents in individual discharges was used to study the nature of these discharges and their distributions in time and position. In order to provide a sufficiently small area, micro-electrodes made of aluminum wires were chose as specimens. Pure aluminum wires (99.99%) with 0.6 mm diameter were anodized in electrolyte, which was composed of commercial distilled water, 5 g/L potassium hydroxide (KOH) and 10 g/L sodium silicate (Na_2_SiO_3_). Experiments were carried out at 25 °C in a 2-liter water-cooled stainless steel tank, which also served as the counter-electrode.

Aluminum wires were first anodized up to DC 550 V at a rate of 8 V/s, to cover the entire metal surface with a thick anodic oxide film. Next, these wires were embedded in an epoxy resin. To expose a clean and relatively flat surface, the embedded wires were sectioned with a slicing knife. All specimens were rinsed under running water to keep the tip surface clean before the next process. 

### 2.2. Single Pulse Anodizing

In our experiments, every single pulse anodizing process was carried out with an individual specimen and the interval time between adjacent experiments was set for 5 min. All single pulse anodizing processes were carried out in two steps. Initially, pre-deposited oxide films on the tip surface were obtained by anodizing with a DC voltage sweep up to 300 V at a rate of 8 V/s. Then, a single pulse voltage with amplitude in the range of 325–525 V and a pulse width of 100–5000 μs was applied to cause dielectric breakdown. 

In addition, a longer voltage pulse with a magnitude at 500 V and pulse duration of 50 ms was applied on these pre-deposited oxide films to estimate electrical characteristic parameters, such as current level, duration, diameter, and spatial distribution ratio. 

### 2.3. Data Monitoring and Post-Processing

For pre-treatment and single pulse anodizing process, voltage and current data were recorded at a sampling rate of 2 MHz using a data acquisition card (National Instruments, PCI-6133, Austin, TX, USA) controlled by the LabVIEW software installed in PC (Thinkpad T580). Current signal was sampled by a hall current sensor (CSM002A) and voltage signal between anode and cathode electrodes was detected by probe of oscilloscope (DPO 3014) directly. Meanwhile, real-time waveforms of current and voltage were displayed on the screen of an oscilloscope. Surfaces of the specimens were observed using a LEO1530 VP scanning electron microscope.

Raw voltage and current data had considerable electrical noise due to mechanical vibration, electron avalanche, dielectric breakdown and high frequency electronic switching. As a result, it was necessary to carry out post-processing for data. The data were first filtered with a low pass filter with a cut-off frequency of 50 kHz. This allowed high frequency noise due to the electronic switching to be eliminated. Next, this data was smoothened using moving average filter. Finally, an appropriate scale function and wavelet basis function were adopted to extract random noise signal. 

## 3. Results

### 3.1. Identification of Critical Breakdown Voltage

**Figure 2:**
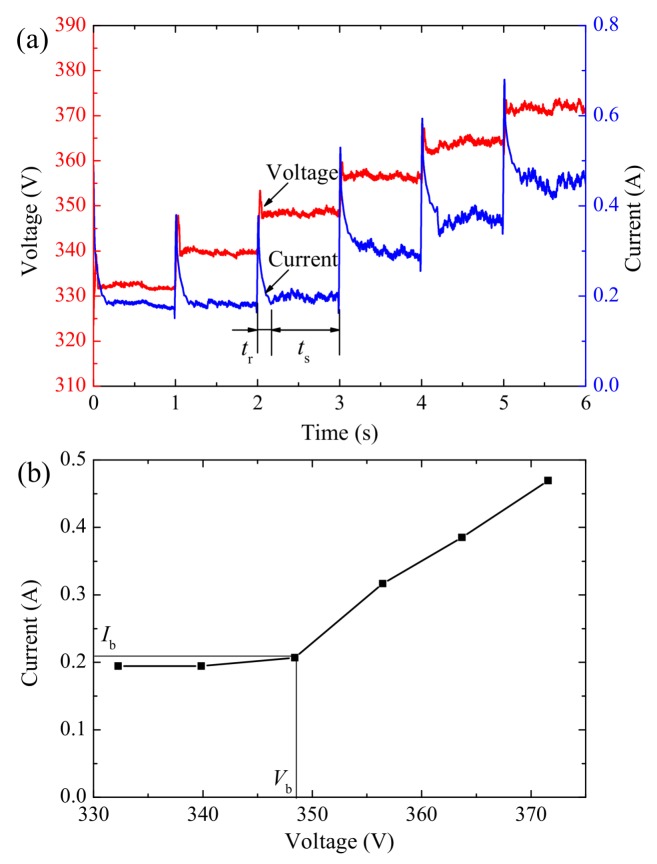
(**a**) Partial voltage and current waveforms during pre-treatment process with voltage sweep at 8 V/s and (**b**) their voltage-current characteristic curve.

Figure 2a shows the partial voltage and current waveforms during the pre-treatment process of aluminum wires. In addition, dielectric breakdown of the oxide films when the potential was increased to approximately 350 V is shown in the figure. Within *t*_r_, a rise of potential results in an abrupt increase in current due to the active capacitive effect of the electrolyte load. After reaching a peak, the current decreases exponentially to a relatively stable level (within *t*_s_). When potential is lower than 340 V, stable levels are almost equivalent to 0.2 A. Above 350 V, the stable level increases with potential with an oscillating current waveform. This probably indicates that dielectric breakdown of oxide films occurred. Before dielectric breakdown, the applied voltage is not high enough to cause discharge and the load keeps in a stable high impedance. So, the current waveform is relatively smooth.

To better understand this phenomenon, appropriate RMS value of the current and voltage approximations were calculated for each step. Stable value of current (within *t*_s_) was chosen to improve approximation accuracy for the voltage-current characteristics shown in Figure 2b.

### 3.2. Electrical Characteristics of Single Pulse Anodizing 

**Figure 3:**
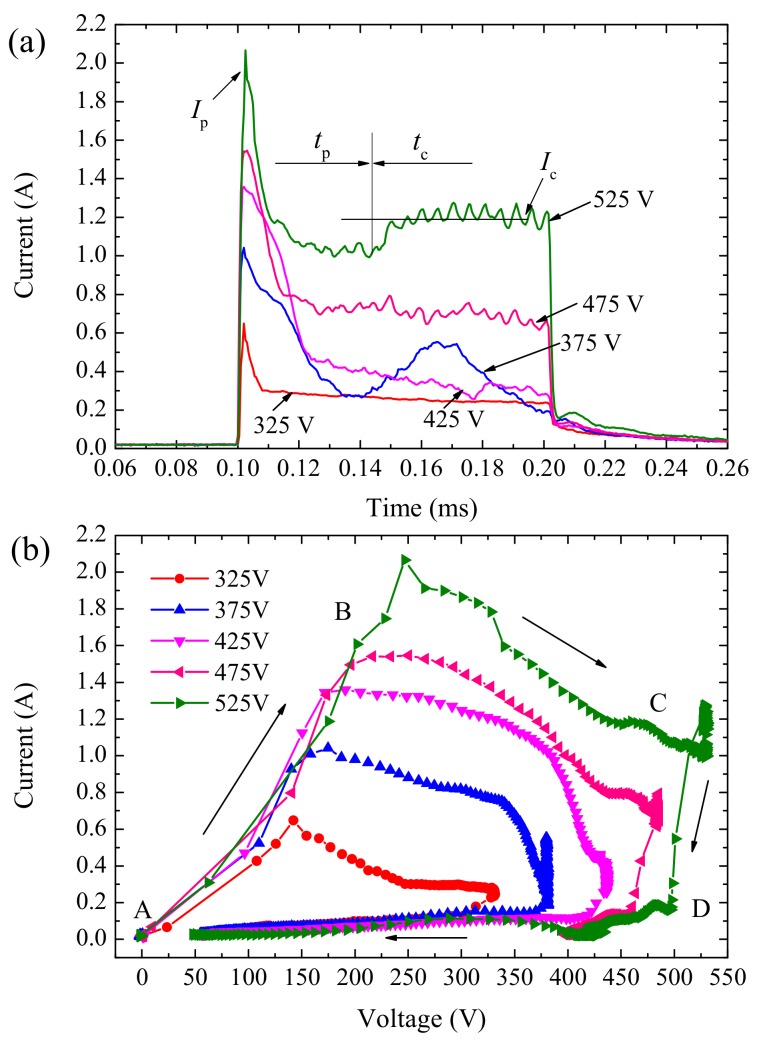
Electrical characteristic curves of single pulse anodizing under different potentials, (**a**) current transients and (**b**) *V*–*I* curve.

To further understand the influence of pulse voltage on electrical characteristics of single pulse anodizing of aluminum micro-electrode, a group of single pulses with different voltages were applied for 100 μs. Figure 3a depicts the current transients due to these pulses. A peak current (*I*_p_) was observed within *t*_p_ and increases with the rise of the pulse voltage. Next, a sharp decrease is seen for the current and it reaches a stable and nearly constant current (*I*_c_) for different level, from 0.2 to 1.2 A, flows within *t*_c_. Peak current due to capacitive load effect produces a large reactive component of the current. Magnitude of *I*_p_ and current transition time *t*_p_ were related to pulse potential. A higher potential resulted in a larger *I*_p_ and longer *t*_p_. An increase in the value of *I*_c_ was also observed with an increase in the applied pulse voltage. However, transient behavior was different at 375 V. Current oscillations with a large amplitude were observed and could be attributed to the tiny flashing sparks.

Figure 3b shows the dynamic *V*–*I* characteristic curves of single pulses, which is characterized by a loop from A to D along the marked direction. An abrupt current increase occurred due to a rapid initial voltage rise (path A-B), which was followed by a decrease in current to a lower level until the voltage reached a target value (path B-C). Next, a gradual and subtle change in voltage and current values were observed (path C-D). Finally, a sudden drop in the current decreases to zero was observed with voltage decreasing at a lower rate (path D-A). Shape and scope of these curves were affected by magnitude and change rate of the pulse potential. Higher potential resulted in longer loop line and larger loop area. 

**Figure 4:**
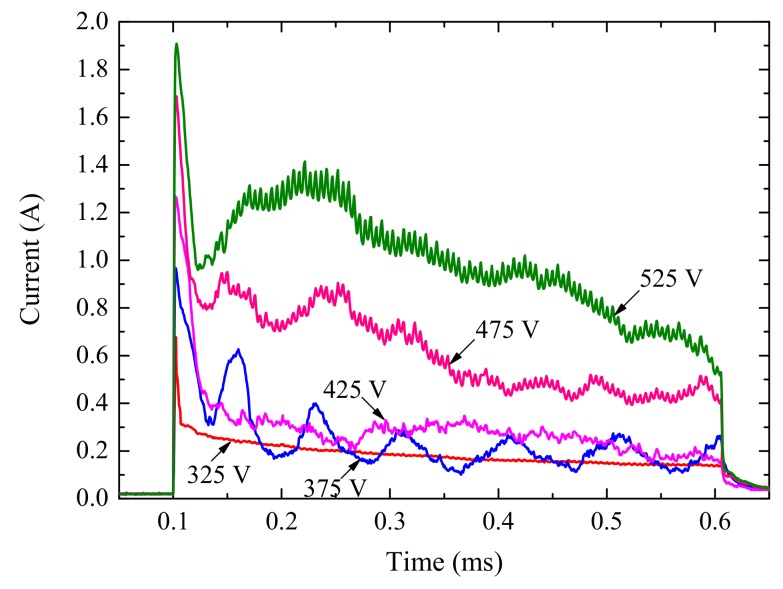
Current transient curves for single pulse anodizing under different potentials.

Similar current transients were observed for pulse voltages ranging from 325 to 525 V with a pulse duration of 500 μs (shown in Figure 4). With an increase in the pulse duration, differences among these pulses were prominent. For the pulse potential lower than 375 V, current transient was relatively smooth and decreased almost linearly with time. When the pulse potential was higher than 375 V, oscillation due to spark discharge was observed in the current transient due to dielectric breakdown of the coatings. For the same pulse potential, the effective value of the pulse current is inversely proportional to the pulse duration. This suggests the existence of a more reactive component in the overall current corresponding to the shorter pulses, due to the initial capacitor charging process. 

### 3.3. Effects of Pulse Parameters on Surface Morphology

**Figure 5:**
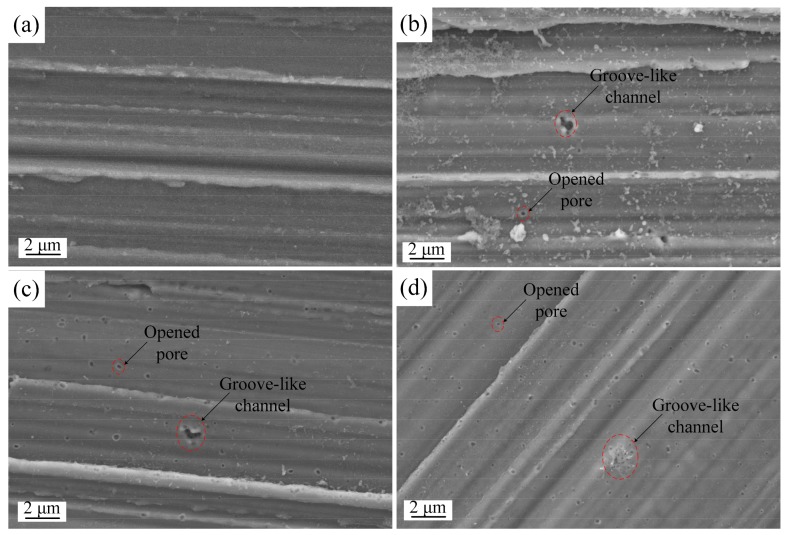
Scanning electron microscope (SEM) images of micro-electrode surfaces, (**a**) before single pulse anodizing, (**b**) anodizing with 375 V/100 μs pulse, (**c**) anodizing with 425 V/100 μs pulse, (**d**) anodizing with 475 V/100 μs pulse.

Surface morphology after single pulse anodizing varies for different pulse voltages. This reflects voltage-dependent discharge characteristics. Figure 5a shows that the surface of the micro-electrode before single pulse anodizing was relatively flat and only several knife marks were visible at a higher resolution. Surfaces of micro-electrodes after single pulse anodizing are shown in Figure 5b–d. Figure 5b shows the surface after anodization using 375 V/100 μs pulse. Similar surfaces were observed in Figure 5c,d, which show anodization surfaces for 425 V/100 μs and 475 V/100 μs, respectively.

**Figure 6:**
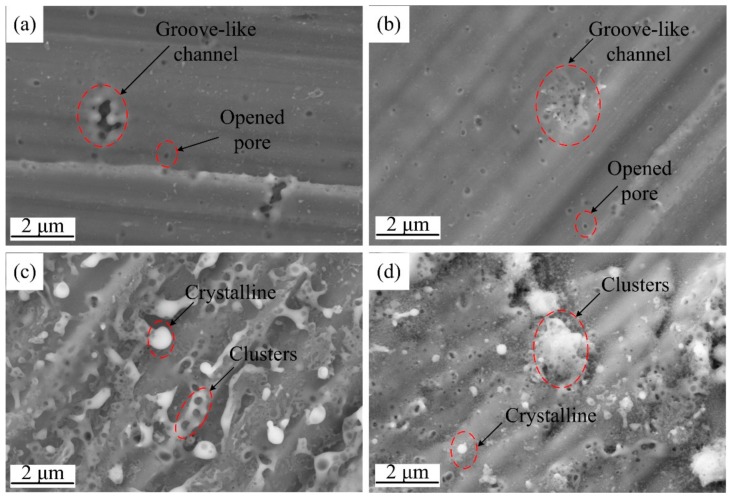
SEM images of micro-electrodes obtained in different conditions, (**a**) anodizing with 400 V/100 μs pulse, (**b**) anodizing with 400 V/500 μs pulse, (**c**) anodizing with 400 V/2 ms pulse, (**d**) anodizing with 400 V/5 ms pulse.

Figure 6 shows the surface SEM images of the specimens after applying a 400 V pulse for different pulse widths. When shorter pulses of 100 μs and 500 μs were applied, circular discharge channels with opened pores were found in addition to the groove-like channels, as shown in Figure 6a,b. These surface morphologies are similar to those shown in Figure 5. However, after breakdown, surface morphology for longer duration pulses was found to be significantly different, as shown in Figure 6c,d. 

### 3.4. Characteristic Parameters of Discharge Event

**Figure 7:**
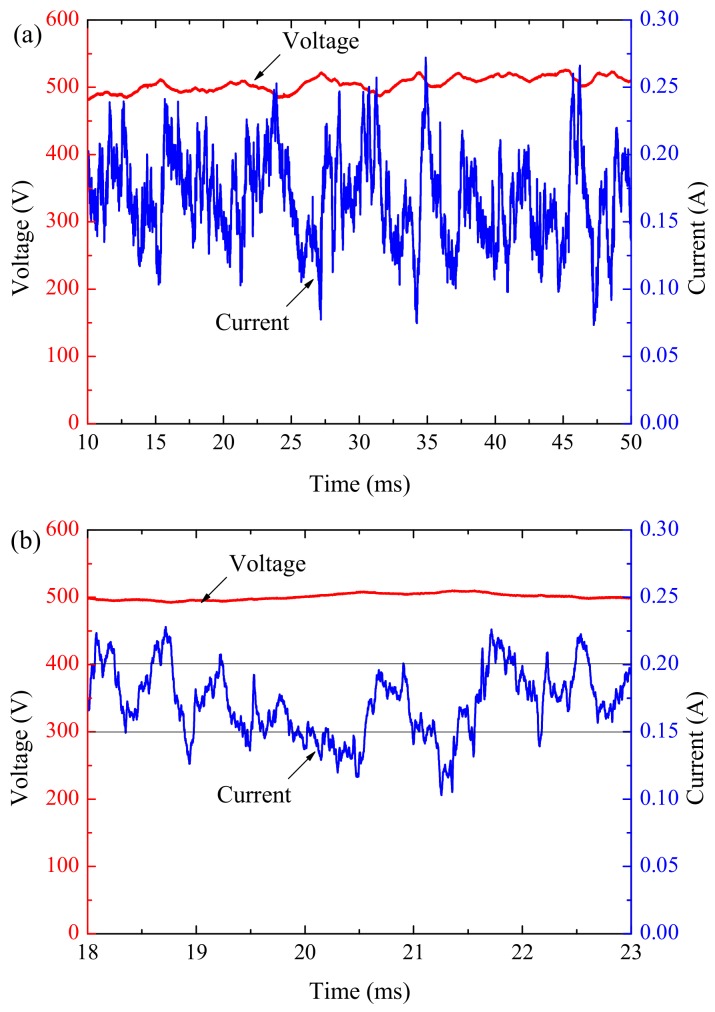
Voltage and current waveforms acquired during anodizing with 500 V/50 ms pulse, at (**a**) low resolution (period of 40 ms) and (**b**) high resolution (period of 5 ms).

Figure 7a shows the raw voltage and current waveforms during anodizing with 500 V/50 ms pulse. Multiple peaks and low baseline were observed in the current waveform. Each peak in the current was accompanied by a decrease in voltage, which recovered during the periods of low current, until the next series of peaks in the current. A magnified section of the current transient is shown in Figure 7b. The discrete nature of individual peaks in the current is evident here. In addition, some cascades appear as the superposition of two current peaks and these probably represent the occurrence of a second discharge event with the first one still ongoing. 

## 4. Discussion

An inflection point can be clearly seen in the voltage-current characteristic curve, as shown in Figure 2b. According to our previous work [26], the voltage value of the inflection point can be considered as the critical breakdown voltage (*V*_b_). Similarly, the corresponding current is the critical breakdown current (*I*_b_). Increase in the current values with increasing voltage suggests breakdown of the anodic oxides occurs continuously for the whole duration. Although breakdown voltage is dependent on layer thickness and some other characteristics, this voltage-current characteristic curve provides a feasible method to identify breakdown voltage of growing oxide film during PEO.

There is a noticeable peak current at the beginning of every single pulse, which was not associated with discharges. This phenomenon appears to be consistent with a hypothesis of active–capacitive load behavior for the PEO process, which was studied systematically in literature [18]. The simple equivalent circuit of PEO load was composed of series-parallel capacitor and resistance, which can have a physical interpretation as the equivalent capacitance of the oxide film and the equivalent resistance introduced by the current passing through the discharges, respectively. The sharp decrease of current was due to the sudden increase of load impedance after the capacitor charging process. In Figure 3a and Figure 4, current oscillations observed at 375 V reflected intense changes of load impedance, caused by dielectric breakdown of oxide film along with abundant randomly flashing sparks. In Section 3.1 and our previous work [26], the initial critical breakdown voltage of oxide film was about 350 V. So, we speculate that the voltage region for these oscillations is among 350–400 V.

It is assumed that electrons cannot flow through the oxide, so the field across it builds up as the applied voltage is raised, and it may reach the breakdown level for the oxide, at which point a discharge occurs [21]. So, the key issue is whether the electric field across the residual oxide film reaches the level necessary for dielectric breakdown. During PEO, the breakdown voltage of oxide film increases with growing layer thickness. In other hand, PEO process can be divided into several stages according to the evolution of the discharges [27]. At the early stage of the process (step 1), the growing oxide film breaks down due to an increase in the applied voltage. This stage is characterized by sparks flashing randomly all over the aluminum alloy surface. Following this stage, sparks progressively change to micro-arcs (step 2). Finally, after the end of the rapid micro-arcs decrease, step 3 is characterized by a few strong remaining arcs that appear on the surface. The average lifetime of the discharges increased with increased voltage and processing time in the main period of PEO coating growth [8]. So, we try to establish the correlation between electrical characteristics and physical phenomena, including the oxide film and discharges. In the single pulse anodizing process, all voltage pulses were applied to the same thin initial oxide film. Pulse energies, which depend on the voltage, current and pulse duration, impact the dielectric breakdown of oxide film and discharge shape significantly. The higher pulse energy results in stronger dielectric breakdown strength and more powerful discharge behavior. 

A comparison of the surface morphology in Figure 5a with Figure 5b–d reveals some distinct changes on the surface of micro-electrodes. Dielectric breakdown due to the single voltage pulse produced a number of isolated discharge channels on this anodic oxide film. Circular opened discharge channels, tens to a few hundreds of nm in size, were found to be surrounded by a band of evidently thicker film material. In addition, large surface regions without discharge pores were also observed. The quantity and size of opened discharge pores increased with an increase in potential. This occurs because with higher electric field strength applied to the oxide film, a larger driving force is available for tunneling ionization. Furthermore, some groove-like discharge channels, which consist of numerous opened and closed discharge pores, were found on the surface, as shown in Figure 5d. Both pores and discharge channels were randomly distributed on the metal surface. This implies the irregular formation of barrier-type anodic oxide films. When numerous narrow pores overlapped in a small region, a groove-like discharge channel was formed. In some case, a few discharge pores were found to be coated with oxide produced by the repeating plasma discharge events during pulse anodizing, resulting in closed pores.

The surface morphology was also found to be dependent on pulse width to some extent. As shown in Figure 6c,d, it can be seen that the entire surface was covered with a thicker layer of anodic oxide film, modified by dielectric breakdown. The oxide surface was rough and a number of bright spots with sizes of ~1 μm or less were observed. Electric field strength between the electrolyte and metal increased with time due to continuous electron transfer. This higher stored energy makes it easier to breakdown the film. Moreover, temperature increase in the groove-like discharge channel regions could result in transforming amorphous alumina to crystalline, which usually has a higher chemical stability [22]. The closure of opened discharge pores appears to be more obvious with increasing pulse width in comparison to pulse magnitude. For both 2 and 5 ms pulse widths, numerous discharge channels were found on the surface. The size of these discharge clusters (circled in Figure 6c,d) on the oxide surface is proportional to the pulse width and an increased size was observed for longer pulse widths.

Some characteristic parameters can be obtained from Figure 7. However, difficulty arises with the identification of the start and end of each discharge event. Using the method mentioned in literature [16], we hypothesized that a peak current (*I*_p_) and a bottom current (*I*_b_) represent a start and end of discharge event respectively. Two current thresholds were set to estimate the total number of discharge events, 0.2 and 0.15 A. When the current exceed 0.2 A, it can be considered as a peak current of a discharge event. When the current value is lower than 0.15 A, it can be considered as the baseline current of a discharge event. Discharges were considered to initiate when the current reaches the peak and terminates when it reaches the baseline current. 

Current level (*I*_event_), duration (*t*_d_), diameter (φ), and spatial distribution ratio (D_n_) of discharge can be estimated using Equations (1)–(6):

(1) $$N_{\mathrm{total}}=\frac{1}{2}\left(N_{p}+N_{b}\right)$$

(2) $$I_{\mathrm{event}}=\overset{N_{\mathrm{total}}}{\underset{n=0}{\sum }}\frac{1}{N_{\mathrm{total}}}\left(I_{p}-I_{b}\right)$$

(3) $$t_{\mathrm{gap}}^{n}=\left|t_{p}^{n}-t_{b}^{n}\right|$$

(4) $$t_{d}=\overset{N_{\mathrm{total}}}{\underset{n=0}{\sum }}\frac{1}{N_{\mathrm{total}}}t_{\mathrm{gap}}^{n}$$

(5) $$D_{n}=N_{\mathrm{total}}/\left(S_{\mathrm{area}}\cdot t_{\mathrm{pulse}}\right)$$

(6) φ=2Sarea(Dn⋅π)

In Equations (1)–(6): N_p_, N_b_ and N_total_ represent the number of *I*_p_, *I*_b_ and total discharge events respectively; *t*_gap_ represents the time interval between adjacent peak time (*t*_p_) and bottom time (*t*_b_); *S*_area_ represents the superficial area of sample; *t*_pulse_ represents the pulse duration. 

To eliminate errors due to experimental deviation, a sample size of ten for the same pulse anodizing was carried out. All the characteristic parameters (shown in Table 1) were obtained by calculating the average value of the results of these experiments.

Although the characteristic parameters listed in Table 1 are similar to those results reported in section ‘1. Introduction’, experimental methods and monitoring techniques need to be improved to acquire more accurate data. In addition, a larger sample size of experiments should be carried out to confirm these results.

## 5. Conclusions

(1) Characteristic phenomenon, with the occurrence of an abrupt current increase when potential reaches a critical value, shown in the voltage-current characteristic curve, allows a feasible identification method for critical breakdown voltage of oxide film.
(2) Magnitude of peak current (*I*_p_), current transition time (*t*_p_), and constant current (*I*_c_) of the single pulse anodizing process were determined by pulse potential. Dynamic V-I characteristic curves for single pulses were characterized by loop circle with the length and area of this loop circle being related to pulse potential. With the increase of pulse width, the effective value of the current becomes smaller.
(3) Characteristic shapes of discharge channels were observed under single pulse anodizing and were correlated with the pulse parameters. Isolated circular opened pores were primarily found under shorter and lower voltage pulses. In contrast, groove-like discharge channels formed in addition to opened pores under longer and higher voltage pulses. Opened discharge pores were found to close within the groove-like discharge channels region with increasing pulse width.
(4) Approximate characteristic parameters of individual discharge events were estimated for individual discharge events of short duration (~hundreds of μs), low current (~tens of mA), and small size (~hundreds of μm).

## 6. Patents

Cao, B.; Yang, K.; Huang Z. Adaptive control method and system for plasma electrolytic oxidation process. *China ZL* 201410036861.5, 2016.

## Figures and Tables

**Table 1:** Estimated characteristic parameters of individual discharge events.

Current Level (*I*_event_, mA)	Duration (*t*_d_, ms)	Spatial Distribution Ratio (D_n_, mm^−2^·ms^−1^)	Diameter (*φ*, μm)
97.1	0.94	4.69	369.4
